# From Calcium Volume Correlations to Whole-Plaque Phenotyping: A Road Still to Be Traveled

**DOI:** 10.1016/j.jacadv.2026.103422

**Published:** 2026-10-30

**Authors:** Andreas S. Papazoglou, Eirinaios Tsiartas, Georgios I. Tagarakis

**Affiliations:** a School of Medicine, Aristotle University of Thessaloniki, Thessaloniki, Greece; b Department of Medicine, University of Cambridge, Cambridge, United Kingdom

We read with great interest the study by van der Waerden et al, who applied artificial intelligence-derived volumetric calcium quantification to optical coherence tomography (OCT) pull backs of nonculprit lesions in post–myocardial infarction patients.^1^ Their findings support a dynamic model: an initial lipid-rich, inflamed phase may be followed by a transitional phase of accumulating vulnerability, and finally by extensive, comparatively inert calcification.

This concurs with existing evidence linking greater calcium burden to lower vascular inflammation and plaque vulnerability,^2^ as well as with the broader coronary calcium paradox, whereby statins and extreme physical activity raise calcium burden while lowering cardiovascular risk. In a combined computed tomography (CT)-OCT imaging study, lipid-rich plaques, macrophages, and microvessels were significantly less prevalent in lesions with high vs intermediate calcium burden.^2^

However, calcium burden alone may not fully capture plaque vulnerability. The biomechanical effects of calcification may depend on its spatial architecture, with the size, orientation, and confluence of calcium deposits determining whether they amplify or shield local mechanical stress. Although van der Waerden et al characterized calcium volume, length, arc, depth, and thickness, the spatial relationship between calcium deposits and surrounding plaque components was not explored. Moreover, the reported associations concern OCT-defined vulnerability features rather than lesion-level clinical outcomes. Whether their greater prevalence at higher calcium burdens translates into greater propensity for future plaque disruption therefore remains uncertain, particularly if calcium architecture also modifies their biomechanical consequences.

Resolving these questions is further complicated by the technical limitations of imaging calcified plaques. OCT excels at superficial, high-resolution morphology but penetrates only 1 to 2 mm, so architecture beneath thick or heavily calcified plaque may be underestimated—a limitation the authors themselves acknowledge, despite their segmentation model's strong performance in calcified plaque. Intravascular ultrasound (IVUS) better captures whole-vessel architecture and deeper components; a combined virtual-histology IVUS-OCT approach could better illuminate the features of interest.^3^ A recent histology-validated hybrid IVUS-OCT deep-learning classifier outperformed single-modality models and expert readers in plaque characterization.^4^

*Ex vivo* micro-CT could provide high-resolution three-dimensional characterization of calcium architecture and, through histopathological validation, help clarify discordant IVUS-OCT findings. In our unpublished work using micro-CT on unstained formalin-fixed, paraffin-embedded carotid plaques, heavy calcification markedly impaired visualization of the fibrous cap, microthrombus, neovessels, and inflammatory infiltrate. Raman spectroscopy further revealed that calcifications of similar volume can differ substantially in crystalline maturity.

Together, these observations raise 2 broader questions: 1) how definitive are conclusions drawn from imaging analyses of soft-tissue features in calcified plaques; and 2) do calcium architecture and composition, not merely quantity, drive plaque vulnerability? Supporting this concept, hydroxyapatite in carotid plaques has been associated with unstable plaques, whereas calcium oxalate has been found mainly in stable plaques, independent of calcium quantity.^5^ These findings highlight the potential value of moving beyond calcium burden toward a more comprehensive calcium phenotype. Hybrid Raman-OCT catheters could characterize the chemical composition of calcified and soft-tissue components alongside morphology, potentially helping explain why volume, length, and arc each capture only part of the vulnerability signal.
